# MicroRNA Monitoring in Human Alveolar Macrophages from Patients with Smoking-Related Lung Diseases: A Preliminary Study

**DOI:** 10.3390/biomedicines12051050

**Published:** 2024-05-09

**Authors:** Davida Mirra, Renata Esposito, Giuseppe Spaziano, Liberata Sportiello, Francesca Panico, Antonio Squillante, Maddalena Falciani, Ida Cerqua, Luca Gallelli, Erika Cione, Bruno D’Agostino

**Affiliations:** 1 Department of Environmental Biological and Pharmaceutical Sciences and Technologies, University of Campania “Luigi Vanvitelli”, 81100 Caserta, Italy; davida.mirra@unicampania.it (D.M.); renata.esposito@unicampania.it (R.E.); bruno.dagostino@unicampania.it (B.D.); 2 Campania Regional Centre for Pharmacovigilance and Pharmacoepidemiology, 80138 Naples, Italy; liberata.sportiello@unicampania.it; 3 Department of Experimental Medicine-Section of Pharmacology “L. Donatelli”, University of Campania “Luigi Vanvitelli”, 80138 Naples, Italy; 4 Department of Health Sciences, University of “Magna Graecia”, 88100 Catanzaro, Italy; francesca.panico@studenti.unicz.it (F.P.); gallelli@unicz.it (L.G.); 5 Department of Medicine, University of Salerno, 84100 Salerno, Italy; a.squillante92@gmail.com; 6 Pulmonary and Critical Care Medicine, Ospedale Scarlato, 84018 Scafati, Italy; m.falciani@aslsalerno.it; 7 Department of Pharmacy, University Federico II of Naples, Via D. Montesano 49, 80131 Naples, Italy; ida.cerqua@unina.it; 8 Department of Pharmacy, Health and Nutritional Sciences, University of Calabria, 87036 Rende, Italy; erika.cione@unical.it

**Keywords:** microRNAs, COPD, alveolar macrophages, smoking-related diseases, lung cancer

## Abstract

Chronic obstructive pulmonary disease (COPD) is a progressive lung disease that is commonly considered to be a potent driver of non-small cell lung cancer (NSCLC) development and related mortality. A growing body of evidence supports a role of the immune system, mainly played by alveolar macrophages (AMs), in key axes regulating the development of COPD or NSCLC phenotypes in response to harmful agents. MicroRNAs (miRNAs) are small non-coding RNAs that influence most biological processes and interfere with several regulatory pathways. The purpose of this study was to assess miRNA expression patterns in patients with COPD, NSCLC, and ever- or never-smoker controls to explore their involvement in smoking-related diseases. Bronchoalveolar lavage (BAL) specimens were collected from a prospective cohort of 43 sex-matched subjects to determine the expressions of hsa-miR-223-5p, 16-5p, 20a-5p, -17-5p, 34a-5p and 106a-5p by RT-PCR. In addition, a bioinformatic analysis of miRNA target genes linked to cancer was performed. Distinct and common miRNA expression levels were identified in each pathological group, suggesting their possible role as an index of NSCLC or COPD microenvironment. Moreover, we identified miRNA targets linked to carcinogenesis using in silico analysis. In conclusion, this study identified miRNA signatures in AMs, allowing us to understand the molecular mechanisms underlying smoking-related conditions and potentially providing new insights for diagnosis or pharmacological treatment.

## 1. Introduction

Chronic obstructive pulmonary disease (COPD) is a fatal disease with high mortality worldwide, placing a heavy economic burden on the healthcare systems [1,2]. It is a heterogenous condition characterized by a progressive deterioration of lung function over time and is generally associated with lung inflammation triggered by harmful particles or gases [3,4,5,6,7,8,9]. Additionally, COPD is commonly considered a potent driver of non-small-cell lung cancer (NSCLC) development and related mortality, beyond a common etiology [10,11]. Indeed, lung cancer is up to five times more frequent in smokers with airflow obstruction than in those with normal lung function, and patients with COPD have twice the risk of cancer diagnosis [12]. Inflammation and tumorigenesis are closely linked, and chronic inflammation could lead to an increased rate of cell division and to the chance of mutations and carcinogenesis [13,14,15,16]. Moreover, a growing body of evidence supports the role of the innate and adaptive immune systems, mainly played by alveolar macrophages (AMs), as key axes that regulate the development of COPD or NSCLC phenotypes in response to harmful agents [17,18,19,20,21,22,23]. Despite this increased attention, the mechanistic nature of both diseases remains unclear, making novel biomarkers necessary to improve the prediction of cancer risk in subjects with COPD. Recently, epigenetic changes, including DNA methylation, covalent histone modifications, and microRNA (miRNA) expression, have been reported to play important roles in lung cancer and COPD onset [24,25]. Indeed, miRNAs are small non-coding, single-stranded RNA molecules capable of binding to mRNA sequences, resulting in gene silencing via mRNA translational repression or target degradation. miRNAs expression can influence most biological processes and interfere with several regulatory pathways, playing a crucial role in the development of both innate and adaptive immune systems [26,27,28,29,30]. In addition, several authors have identified serum miRNAs, which could serve as biomarkers to help identify the risk of developing cancer in COPD patients [31,32]. In the present study, we focused on miRNAs that have recently been shown to influence immune system regulation or cancer hallmarks, such as cell proliferation and immunosuppression. Notably, hsa-miR-34a-5p has been reported to be dysregulated in various cancers and considered a tumor suppressor because of its synergistic effect with p53 [33]. Hsa-miR-223-5p was found to be downregulated in the bronchial airway of lung cancer patients and regulated the malignant phenotypes of lung squamous cell carcinoma cells by pairing with OTX1 [34]. Similarly, hsa-miR-16-5p is a tumor inhibitor and a new biomarker for immune-checkpoint-inhibitor-dependent immunotherapy in lung adenocarcinoma by regulating the PD-L1 expression [35]. Furthermore, different authors have shown that hsa-miR-106a-5p is overexpressed in the sera of patients with NSCLC [36] while hsa-miR-20a-5p and hsa-miR-17-5p have been reported as tumor suppressors [37,38,39]. However, miRNAs could be shared in COPD and cancer targeting candidate mRNA families implicated in the pathogenesis of both diseases [40]). Therefore, to obtain miRNA signatures between COPD and NSCLC that could be used to distinguish between smoking-related diseases, we evaluated miRNAs expression in AMs of bronchoalveolar lavage (BAL) from patients with COPD, NSCLC, and ever- or never- smoker controls. 

## 2. Materials and Methods

### 2.1. Study Population

We performed an observational clinical study on 43 age- and sex-matched subjects, aged 18 years or older, referred to the “Mater Domini” Hospital in Catanzaro, Italy, with a suspected diagnosis of pulmonary neoplasia, undergoing routine bronchoscopy and bronchoalveolar lavage (BAL). This study is a part of the clinical trial recorded at clinicaltrials.gov (NCT04654104) and approved by the local Ethics Committee “Calabria Centro” (n. 361 of 22 October 2020). This study was conducted in compliance with the Institutional Review Board/Human Subjects Research Committee, the Declaration of Helsinki, and the Guidelines for Good Clinical Practice criteria. Before the beginning of the study, all the enrolled patients or their legal guardians signed their informed consent and underwent spirometry according to international guidelines [41]. At enrollment, patients’ demographics and clinical and social history were obtained by physicians: age, gender, smoking history (pack/years), spirometry data, comorbidities, and pharmacological treatments. The exclusion criteria were active pulmonary infections, autoimmune diseases, extrapulmonary neoplasia, or other airflow obstruction, such as asthma or bronchiectasis, or those who did not sign their informed consent. The main clinical and pathological characteristics of cohorts are summarized in Table 1. The enrolled subjects were classified into four groups according to their clinical and pathological (bronchoscopy-guided) diagnoses: (1) healthy never-smokers control (“HNS”; *n* = 9); (2) healthy ever-smokers control (“HS”; *n* = 11); (3) smokers with Global Initiative for Obstructive Lung Diseases (GOLD) stage 1–4 (“COPD”; *n* = 11); and (4) non-small-cell lung cancer (“NSCLC”; *n* = 12). Within each group, subjects were comparable with respect to COPD severity and/or lung cancer histology. Indeed, only those with NSCLC were enrolled among the subjects presenting cancer. The most frequent comorbidities were hypertension and diabetes mellitus; bronchodilators were the most common medications used.

### 2.2. Bronchoalveolar Lavage

All subjects underwent bronchoscopy for clinical indications, and BAL was obtained with a bronchoscope according to international guidelines [41] The procedure involved premedication (20 mg codeine per os) and local anesthesia of the larynx and lower airways (0.5% tetracaine in the oropharynx, 8 cc 0.5% tetracaine in lower airways). Transcutaneous oxygen saturation was monitored continuously through an oximeter with a finger probe. BAL was obtained by instilling a total volume of 200 mL of sterile isotonic saline solution (NaCl 0.9%, 37 °C) in the right middle lobe. Specifically, fraction I, returned after instilling 50 mL of saline, and fraction II, returned after instilling 3 × 50 mL of saline, were collected in a siliconized specimen trap, and immediately kept on ice. BAL was filtered through nylon gauze and centrifuged (10 min at 400× *g* at 4 °C). The cell pellet was washed twice, counted, and resuspended in PBS. Cells were counted in a Bürker chamber and cell yield was determined by the total cell number per fraction/total recovered volume per fraction. Cell viability was determined by Trypan blue and a differential cell count was performed by microscopy on the cytospin slide after staining with QUICK-DIFF KIT (Reastain^®^, Reagena Toivala, Finland); at least 100 cells were counted. AMs were identified as greater than 95% by morphology. 

### 2.3. Clinical Biochemistry Assays and Real Time PCR (RT-PCR)

To extract miRNAs (total RNA) from AMs from BAL, we adopted the best method available: the miRNeasy Mini kit (QIAGEN, Hilden Germany), used according to manufacturer instructions. RNA was eluted at a volume of 15 µL with RNAse-free water. The qubit RNA Integrity and Quality (IQ) assay (catalog number Q33222) was performed to check for RNA degradation, and then microRNAs were quantified with a Qubit™ microRNA assay kit (catalog number Q 32881). For both kits, a Qubit 4 fluorometer (Thermo Fisher Scientific, Waltham, MA, USA) (serial number 2322618032114) was used.

The expression levels of hsa-miR-223-5p, 16-5p, 20a-5p, -17-5p, 34a-5p, and 106a-5p were determined through TaqMan™ Advanced miRNA Assay RT-PCR, following the Thermo Fisher Scientific procedures (Waltham, MA, USA) and selecting U6 snRNA as the housekeeping miRNA (Gallelli et al., 2019). The sequences used were:

- hsa-miR-223-5p: CGUGUAUUUGACAAGCUGAGUU; (Assay ID 477984_mir);
- hsa-miR-16-5p: UAGCAGCACGUAAAUAUUGGCG; (Assay ID 477860_mir);
- hsa-miR-20a-5p: UAAAGUGCUUAUAGUGCAGGUAG; (Assay ID 478317_mir);
- hsa-miR-17-5p: CAAAGUGCUUACAGUGCAGGUAG; (Assay ID 478447_mir);
- hsa-miR-34a-5p: UGGCAGUGUCUUAGCUGGUUGU; (Assay ID 478048_mir);
- hsa-miR-106a-5p: AAAAGUGCUUACAGUGCAGGUAG; (Assay ID 478225_mir);
- U6 snRNA: GTGCTCGCTTCGGCAGCACATATACTAAAATTGGAACGATACAGAGAAGATTAGCATGGCCCCTGCGCAAGGATGACACGCAAATTCGTGAAGCGTTCCATATTTT; (AssayID: 001973).

Briefly, 5–10 ng of total RNA was subjected to a reverse transcription polymerase chain reaction using the TaqMan MicroRNA Reverse Transcription kit for all the miRNA targets chosen, according to the manufacturer’s instructions. The thermocycling conditions were as follows: 42 °C for 30 min, 85 °C for 5 min and 4 °C for 5 min. RT-PCR was performed using the TaqMan™ Fast Advanced Master Mix (Thermo Fisher Scientific, Waltham, MA, USA) according to the manufacturer’s protocol and QuantumStudio5TM Real-Time PCR Systems. The thermocycling conditions were as follows: 95 °C for 10 min and 40 cycles of 15 s at 95 °C, followed by 1 min at 60 °C. Nine biological replicates for the HNS group, eleven for HS, eleven for NSCLC, and twelve for COPD were analyzed, and all samples were run in triplicate; after the achievement of the RT-PCR, the cycle threshold (Ct) of the reactions was determined. Gene expression stability was evaluated using RefFinder, a web tool integrating geNorm, Normfinder, BestKeeper, and the comparative ΔΔCt method. Data from all RT–PCR experiments and miRNA expression were analyzed by normalizing to the endogenous miRNA control, applying the comparative 2^−ΔΔCt^ method, where ΔCt = Ct_miRNA_ − Ct _housekeeping miRNA_, whereas the relative quantification of differences in expression was conducted with ΔΔCt = ΔCt_HS/COPD/NSCLC_ − ΔCt_HNS_.

### 2.4. In Silico Analysis

To identify genes as targets of hsa-miR-223-5p, 16-5p, 20a-5p, -17-5p, 34a-5p, and 106a-5p linked to NSCLC and/or COPD, we performed in silico analysis. The in silico identification of the target genes was performed using DIANA Tools (http://diana.imis.athena-innovation.gr/DianaTools/index.php, accessed on 8 January 2024) and miRTargetLink 2.0 (https://ccb-compute.cs.uni-saarland.de/mirtargetlink2 accessed on 10 January 2024) databases. These databases were used to check which miRNA target genes were already validated experimentally. Indeed, all mRNAs’ targets chosen were selected from those validated by techniques such as immunoprecipitation, qPCR, or Western blotting. Possible biochemical pathways were checked using the GeneCard database (https://www.genecards.org/ accessed on18 January 2024), allowing genomic, proteomic, transcriptomic, genetic, and functional information on all known and predicted human genes. 

### 2.5. Statistical Analysis

Unless specified, all data are expressed as mean ± standard deviation (SD). The one-way ANOVA test followed by Tukey’s Multiple Comparison Test was used to evaluate the differences between the groups. We used nominal (sex, comorbidity, and treatment) and categorical variables and the correlation between clinical characteristics was calculated using one-way ANOVA followed by Tukey’s Multiple Comparison Test. GraphPad software (version 9.1.0) was used for statistical analyses (GraphPad Software, San Diego, CA, USA). Differences were considered statistically significant at *p* < 0.05.

## 3. Results

### 3.1. miRNA Expression Levels

#### 3.1.1. hsa-miR-223-5p

**Figure 1:**
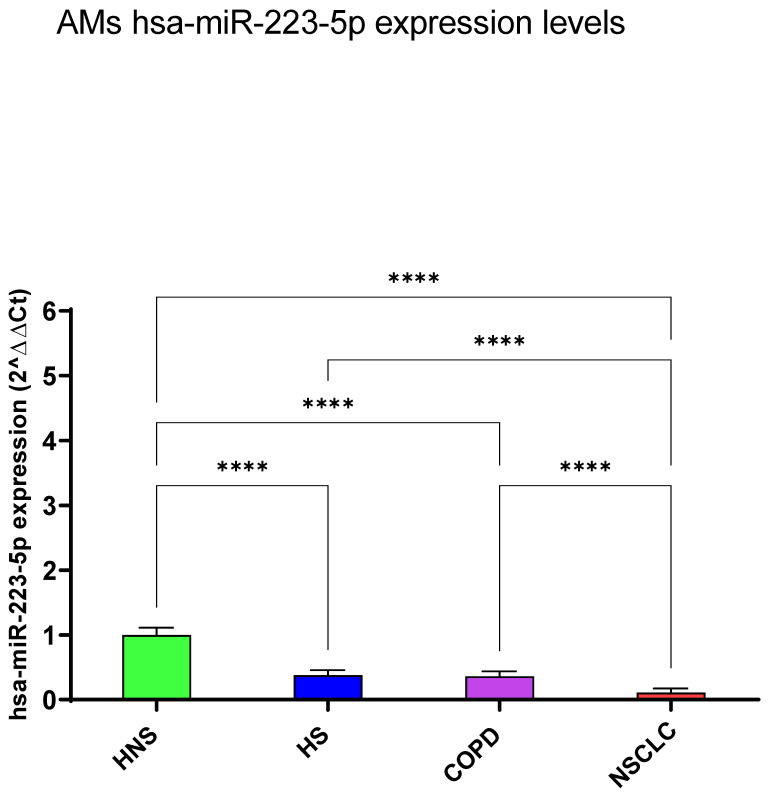
Analysis of hsa-miR-223-5p AMs expression levels in HNS (green column: biological replicates *n* = 9), HS (blue column: biological replicates *n* = 11), COPD (purple column: biological replicates *n* = 11) and NSCLC (red column: biological replicates *n* = 12). All samples were run in triplicate, and results are shown as means ± SD. The statistical tests used in these analyses were one-way analysis of variance followed by Tukey’s Multiple Comparison Test. **** *p* < 0.0001.

We identified common and discriminating miRNA signatures for each pathological entity (NSCLC and COPD) or smoking habit (HS) compared to the control group (HNS). RT-PCR analysis indicated that HS, COPD, and NSCLC shared a significant negative modulation of hsa-miR-223-5p expression levels compared to HNS (*p* < 0.0001, respectively) (Figure 1). Specifically, among the three groups, NSCLC showed the lowest values (*p* < 0.0001, respectively) (Figure 1). 

#### 3.1.2. hsa-miR-16-5p and 20a-5p

**Figure 2:**
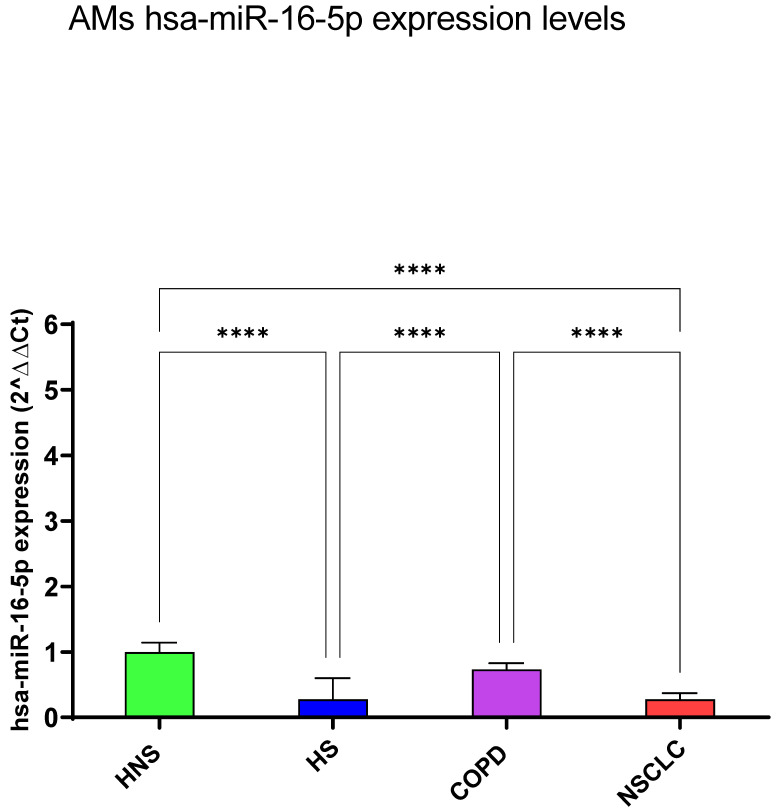
Analysis of hsa-miR-16-5p AMs expression levels in HNS (green column: biological replicates *n* = 9), HS (blue column: biological replicates *n* = 11), COPD (purple column: biological replicates *n* = 11) and NSCLC (red column: biological replicates *n* = 12). All samples were run in triplicate, and results are shown as means ± SD. The statistical tests used in these analyses were one-way analysis of variance followed by Tukey’s Multiple Comparison Test. **** *p* < 0.0001.

**Figure 3:**
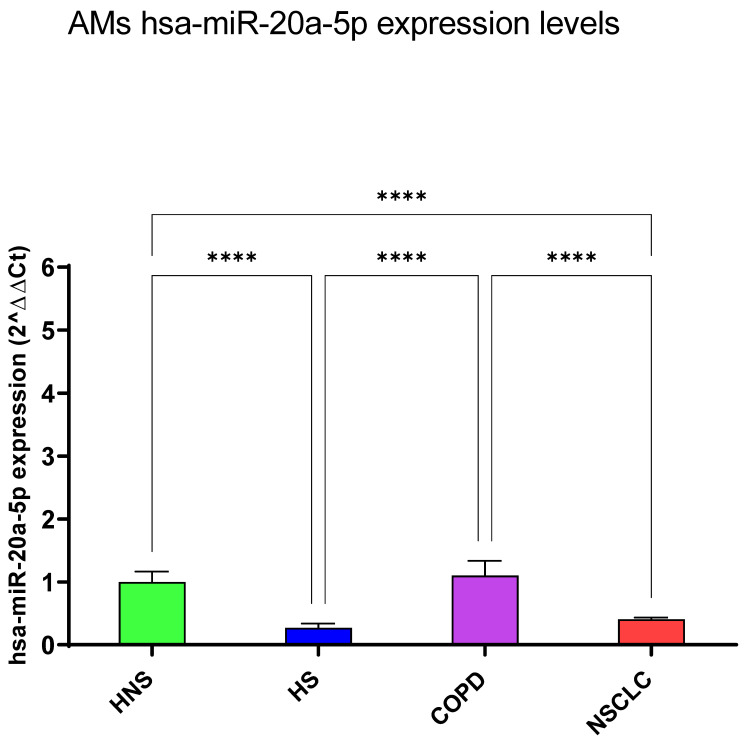
Analysis of hsa-miR-20a-5p AMs expression levels in HNS (green column: biological replicates *n* = 9), HS (blue column: biological replicates *n* = 11), COPD (purple column: biological replicates *n* = 11) and NSCLC (red column: biological replicates *n* = 12). All samples were run in triplicate, and results are shown as means ± SD. The statistical tests used in these analyses were one-way analysis of variance followed by Tukey’s Multiple Comparison Test. **** *p* < 0.0001.

hsa-miR-16-5p and 20a-5p levels were significantly reduced in HS and NSCLC patients with respect to HNS (*p* < 0.0001). Moreover, the expression levels of these miRNAs in COPD patients showed no significant differences from healthy subjects (Figure 2 and Figure 3). 

#### 3.1.3. hsa-miR-17-5p

**Figure 4:**
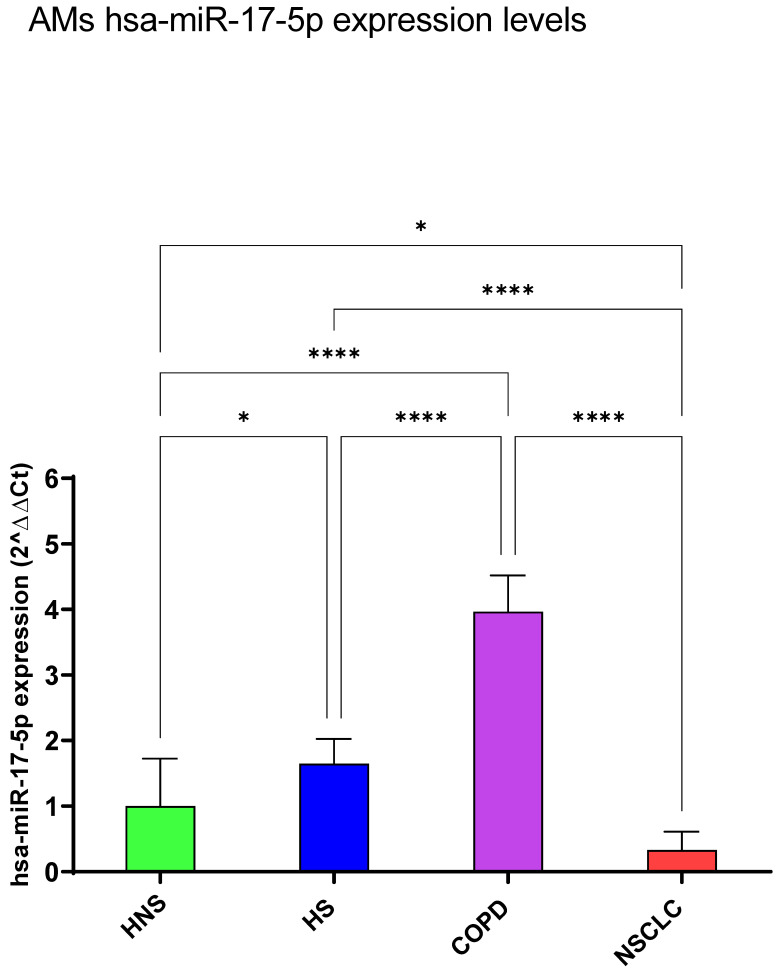
Analysis of hsa-miR-17-5p AMs expression levels in HNS (green column: biological replicates *n* = 9), HS (blue column: biological replicates *n* = 11), COPD (purple column: biological replicates *n* = 11) and NSCLC (red column: biological replicates *n* = 12). All samples were run in triplicate, and results are shown as means ± SD. The statistical tests used in these analyses were one-way analysis of variance followed by Tukey’s Multiple Comparison Test. * *p* < 0.05; **** *p* < 0.0001.

HS and COPD shared a significantly positive modulation of hsa-miR-17-5p expression levels compared to HNS (*p* < 0.05 and *p* < 0.0001, respectively) and particularly to NSCLC (*p* < 0.0001, respectively), in which a significant downregulation was observed with respect to healthy subjects (*p* < 0.05) (Figure 4).

#### 3.1.4. hsa-miR-34a-5p

**Figure 5:**
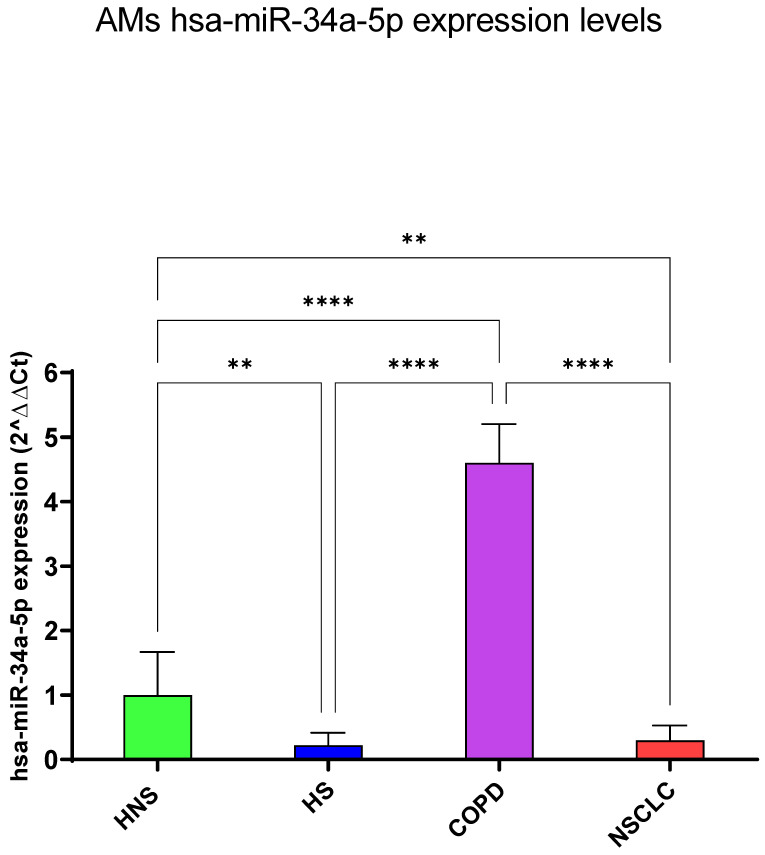
Analysis of hsa-miR-34a-5p AMs expression levels in HNS (green column: biological replicates *n* = 9), HS (blue column: biological replicates *n* = 11), COPD (purple column: biological replicates *n* = 11) and NSCLC (red column: biological replicates *n* = 12). All samples were run in triplicate, and results are shown as means ± SD. The statistical tests used in these analyses were one-way analysis of variance followed by Tukey’s Multiple Comparison Test. ** *p* < 0.01; **** *p* < 0.0001.

Interestingly, RT-PCR results showed that hsa-miR-34a-5p was exclusively upregulated in patients with COPD (*p* < 0.0001), while HS and NSCLC shared a significant negative modulation of this miRNA compared to HNS (*p* < 0.01, respectively) (Figure 5). 

#### 3.1.5. hsa-miR-106a-5p

**Figure 6:**
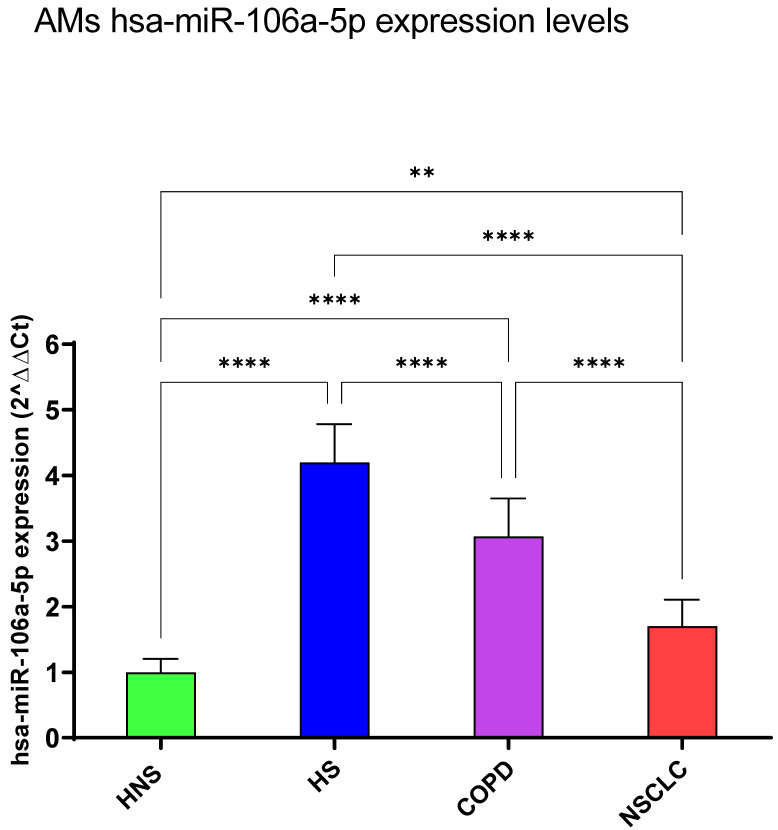
Analysis of hsa-miR-106a-5p AMs expression levels in HNS (green column: biological replicates *n* = 9), HS (blue column: biological replicates *n* = 11), COPD (purple column: biological replicates *n* = 11) and NSCLC (red column: biological replicates *n* = 12). All samples were run in triplicate, and results are shown as means ± SD. The statistical tests used in these analyses were one-way analysis of variance followed by Tukey’s Multiple Comparison Test. ** *p* < 0.01; **** *p* < 0.0001.

The three pathological groups shared upregulated hsa-miR-106a-5p expression levels as compared with the control group (*p* < 0.0001 for HS and COPD group, *p* < 0.01 for NSCLC group), with the highest levels observed in HS subjects versus COPD and NSCLC groups (*p* < 0.0001, respectively) (Figure 6).

### 3.2. In Silico Identification of Target mRNAs

To assess the potential interconnection of miRNAs discriminating each pathological entity studied, we performed in silico prediction analysis based on sequence similarity between miRNAs and mRNAs. Two different databases were used for the analysis and data were compared with respect to the number of target genes experimentally validated. The results are reported in Table 2. The numbers of validated target genes were higher in miR target Link 2.0; therefore, it was used for bioinformatics analysis. We analyzed all selected miRNA targets, focusing on those that are common to several miRNAs and implicated in the cellular pathways responsible for carcinogenesis. The results showed that miRNA may be involved in the regulation of several lung cancer driver genes, such as BCL2, CYCS, E2F2, MCL1, or MYC, among others. However, other target genes linked to tumor invasion and metastasis were found, as shown in Table 3. Given that several authors have experimentally confirmed all genes, miRNAs might modulate these targets in a coordinated or individual manner, affecting several hallmarks of lung carcinogenesis. Abbreviations, names of the selected genes, methods, tissues of validation, and references are reported in Table 4.

## 4. Discussion

In this study, we assessed the role of miRNAs in two smoking-related diseases, COPD and NSCLC, by discriminating between common and selectively dysregulated miRNAs associated with each condition or smoking habit. NSCLC and COPD are lung diseases that share etiological and pathogenic factors [10,11,12]. Indeed, inflammation and tumorigenesis are closely linked, and chronic inflammation in COPD can lead to an increased rate of cell division and the chance of mutations and carcinogenesis [13,14,15,16]. Given the high prevalence of lung cancer in COPD patients, the search for new biomarkers has prompted in-depth epidemiological studies [42,43]. Here, we analyzed a miRNA panel identifying miRNAs that may be potentially useful as biomarkers or for therapeutic purposes. This analysis was carried out in AMs recovered from BAL, a precious biological sample that is highly representative of the pulmonary microenvironment [44]. First, we identified two similarly dysregulated miRNAs in HS, COPD patients, and NSCLC patients, suggesting common pathological mechanisms underlying these conditions. Specifically, hsa-miR-223-5p levels were decreased and hsa-miR-106a-5p levels were increased in these groups compared with those in healthy never-smokers. hsa-miR-223-5p was often reported to be downregulated in NSCLC tissue and cells, leading in turn to the upregulation of many target genes that contribute to tumor proliferation, migration, and invasion [45,46]. In addition, our findings in the ever-smokers and COPD group suggested that hsa-miR-223-5p expression could indicate a common pathway between COPD and NSCLC, probably sustained by cigarette smoke exposure. Several authors have described the role of hsa-miR-223-5p in macrophage differentiation, neutrophil recruitment, and pro-inflammatory responses, which are the key features of lung inflammation and remodeling [47]. For instance, Schembri et al. reported lower hsa-miR-223-5p expression levels in bronchial epithelial cells from current smokers than in those from never-smokers [48]. BTG3, a member of the B-cell translocation gene/transducer of the ErbB2 antiproliferative protein family, which regulates cell cycle progression and differentiation in various cell types, has been reported to be downregulated in NSCLC specimens and cell lines, with the upregulation of hsa-miR-106a-5p [49]. Furthermore, Ye et al. showed that hsa-miR-106a-5p is overexpressed in the serum of patients with NSCLC compared to healthy subjects, thus playing a pivotal role in the onset and development of the disease [36]. Consistent with these data, we observed a significant positive modulation of hsa-miR-106a-5p in the NSCLC, COPD, and HS groups compared to healthy individuals, thus providing evidence of cancer-like dysregulation in COPD and smoking habits. Although a few studies have investigated hsa-miR-106a-5p expression patterns associated with cigarette smoke and chronic lung diseases, Sharma et al. reported that it negatively regulates IL-10 expression in an in vitro and in vivo model of airway inflammation, which is in accordance with our data [50]. Interestingly, COPD exhibited different trends in hsa-miR-16-5p, 20a-5p, and 34a-5p expression compared to HS and NSCLC. This evidence suggests a crucial role for cigarette smoking in the mechanisms responsible for the development of a cancer-like milieu, and that the dysregulation of these miRNAs in COPD subjects towards the levels found in NSCLC could be indicative of an overlap between the two phenotypes. Programmed cell death ligand-1 (PDL1) is a well-known immune checkpoint that is upregulated in patients with lung cancer, allowing tumors to escape immune attack, cell growth, and metastasis [51]. Chen et al. reported that the overexpression of PD-L1 accelerated tumor growth and decreased exosomal hsa-miR-16-5p content in cell culture media, while exosomal miR-16-5p overexpression in cell culture media inhibited tumor development by decreasing PD-L1 expression [35]. Wei et al. reported a 5.5-fold suppressed expression of hsa-miR-16-5p in lung cancer tissues in comparison to the normal adjacent tissues [52]. Consistent with these data, we observed a significant decrease in hsa-miR-16-5p in both HS and NSCLC patients with respect to subjects with COPD. Similarly, hsa-miR-20a-5p showed a different trend between ever-smokers and NSCLC patients compared with COPD subjects. Angiogenesis is an essential feature of carcinogenesis, involving molecules in the lung microenvironment that recruit endothelial cells to promote the formation of intra-tumoral capillaries and primary tumor growth, proliferation, and metastasis [53]. Among these molecules, RRM2 plays a crucial regulatory role in controlling intratumoral capillary formation through the PI3K/Akt axis [54]. Han et al. found that the expression of RRM2 was upregulated, whereas the expression of hsa-miR-20a-5p was downregulated in cancer tissues compared to adjacent tissues in NSCLC patients, identifying hsa-miR-20a-5p as a potential tumor suppressor [37]. Accordingly, our findings showed a negative modulation of hsa-miR-20a-5p not only in the NSCLC group but also in ever-smokers, suggesting the ability of cigarette smoke to modulate its expression in a cancer-like manner. hsa-miR-34a-5p has been extensively evaluated in NSCLC and is commonly considered a tumor suppressor because of its modulation of cellular proliferation, apoptosis, and immune evasion [33]. In a previous study, we reported that NSCLC and COPD patients differed in hsa-miR-34a-5p lung tissue levels, showing an increase and decrease, respectively, compared to healthy controls [55]. In line with these observations, AMs recovered from the BAL of lung cancer and COPD subjects showed an opposite trend in hsa-miR-34a-5p levels. Moreover, there were no statistical differences between ever-smokers and patients with lung cancer, suggesting a similar hsa-miR-34a-5p modulation. Cigarette smoking directly damages bronchial epithelial cells, the first barrier for the respiratory tract, and leads to the infiltration of immune cells into the lung tissue, including macrophages and neutrophils, that sustain inflammation [56]. SIRPα, a member of the immunoglobulin superfamily, modulates many aspects of the inflammatory response, including activation, chemotaxis, and phagocytosis [57]. In macrophages, the upregulation of hsa-miR-17-5p by lipopolysaccharides (LPS) serves as the mechanism underlying LPS-induced SIRPα reduction and macrophage activation [58]. This was consistent with our finding of higher hsa-miR-17-5p levels in both COPD patients and ever-smokers, supporting the importance of smoking in the mechanisms underlying COPD. Intriguingly, NSCLC showed the lowest levels of hsa-miR-17-5p, in accordance with several studies that reported it as a tumor suppressor [38,39] However, hsa-miR-17-5p’s regulatory effects on tumor progression are different and cannot be generalized, making its role controversial [59]. It is important to point out that although all subjects with COPD were current smokers, our data revealed that hsa-miR-16-5p, 20-5p, and 34a-p expression levels differed from those in ever-smokers. Generally, the intensity of the reaction against immunogenic antigens produced in response to cigarette smoke varies across a wide range of disease manifestations, such as COPD or lung cancer. Moreover, recent studies have highlighted the crucial role of immune responses in the regulation of the development of COPD or NSCLC phenotypes in response to smoke, assuming that COPD may be associated with a shift from an innate response to an adaptive immune response with hallmarks typical of autoimmune processes [60,61]. In this context, the dysregulation of these specific miRNAs could reflect phenotype switching or the onset of different lung manifestations, underlining the prominent but not exclusive role of cigarette smoking. Indeed, miRNA expression profiles could be confounded by other environmental conditions, which could further modulate the correlation between the expression of miRNAs and their target mRNAs in COPD [62]. Finally, to assess the potential interconnection of miRNAs discriminating each pathological entity, we performed in silico prediction of hsa-miR target genes. Bioinformatic prediction results revealed that the miRNAs analyzed may potentially be involved in the regulation of several lung cancer driver genes, such as BCL2, CYCS, E2F3, MCL1, MFN2, MYC, RNF115, SENP1, SLC1A5, SLC6A4, SLC7A11, and TMOD3, which are involved in cancer key regulatory pathways [63,64,65,66,67,68,69,70,71,72,73,74,75]. Since several authors have experimentally confirmed all miRNA-regulated genes, miRNAs may modulate these targets in combination or individually, affecting different hallmarks of lung carcinogenesis. For instance, Lu et al. assessed the regulation of hsa-miR-17-5p on MFN2 expression using a luciferase reporter assay system. The authors reported that hsa-miR-17-5p regulates proliferation and apoptosis in human pulmonary artery smooth muscle cells (hPASMCs) treated with hypoxia through MFN2 modulation and binding to its 3′-UTR [76]. Christoffersen et al. showed that the 3′-UTR sequence of MYC revealed the presence of a perfectly complementary 6-nucleotide seed match to hsa-miR-34a-5p. Western blotting revealed that the overexpression of hsa-miR-34a-5 p resulted in a marked downregulation of endogenous MYC protein, emphasizing its significance as a tumor suppressor [77]. 

## 5. Conclusions

Our study identified distinct miRNAs in AMs obtained from BAL samples that could discriminate between smoking-related diseases like NSCLC and COPD. One potential limitation was the small sample size used for miRNA analysis, which did not allow our findings to be applied to a broader population or for a subanalysis to be performed according to COPD severity. Moreover, it might be interesting in the future to analyze the direct effect of smoke in the correlation between miRNAs and their targets linked to carcinogenesis, as well as the interplay between AMs and structural alveolar cells. However, considering the nature of the lung samples used, our results, even if preliminary, could be of clinical relevance and lead to future studies involving larger populations. Our data on common and specific deregulated miRNAs in NSCLC and COPD could allow us to understand the underlying regulatory mechanisms involved in the pathogenesis of these smoking-related conditions and potentially provide new tools for diagnosis or pharmacological treatment.

## Figures and Tables

**Table 1:** Demographic characteristics of the enrolled patients. Data are means ± SD unless specified. The statistical tests used in these analyses were one-way ANOVA followed by the Tukey Multiple Comparison Test. FEV1: forced expiratory volume in the first second; FVC, forced vital capacity; LABA: long-acting beta-agonists; SABA: short-acting beta-agonists; LAMA: long-acting muscarinic agents.

	Healthy Never-Smokers	Healthy Smokers	COPD	NSCLC	*p*-Value
**Total Participants (N)**	9	11	11	12	
**Age (SD)**	70.5 (15.2)	52.5 (8.3)	63.5 (6)	75 (16.9)	NS
**Gender (M/F)**	5/4	6/5	5/6	6/6	NS
**Smoking history (pack/years)**	NA	35	28.3	31	<0.001
**Smoking habit** **(current/former smoker)**	NA	8/3	11/0	6/6	0.0133
**FEV1 (% predicted)**	96% (3.2)	91% (12)	66% (14)	88% (11)	0.0438
**FEV/FVC**	76.1 (2.5)	75 (1.4)	59 (1.7)	74 (3)	<0.0001
**Comorbidities**					
**Hypertension (%)**	5 (55.5%)	5 (45.4%)	3 (27.2%)	5 (41.6%)	0.0152
**Other cardiovascular diseases (%)**	2 (22.2%)	0	3 (27.2%)	1 (12.5%)	NS
**Diabetes mellitus (%)**	1 (16.7%)	0	0	1 (0.8%)	NS
**Medications**					
**Inhaled corticosteroids (N, %)**	0	0	0	0	NS
**LABA/SABA/LAMA (N, %)**	0	0	11 (100%)	3 (25%)	<0.0001

**Table 2:** Bioinformatics tools for in silico analysis. Number of validated genes for each miRNA analyzed in miR Target Link 2.0 and Diana Tools databases.

Number of Target Genes		
miRNA	miR Target Link 2.0	DIANA Tools
hsa-miR-223-5p	551	10
hsa-miR-16-5p	2279	455
hsa-miR-20a-5p	1659	611
hsa-miR-17-5p	1817	136
hsa-miR-34a-5p	968	324
hsa-miR-106a-5p	1166	435

**Table 3:** miRNA gene interaction and possible biochemical pathways involved.

Biochemical Pathways	miRNA	Validated Target Genes
DNA replication—apoptosis	hsa-miR-16-5p hsa-miR-17-5p hsa-miR-20a-5p hsa-miR-34a-5p	BCL2
DNA damage telomere stress—senescence pathways	hsa-miR-16-5p hsa-miR-17-5p hsa-miR-20a-5p hsa-miR-34a-5p	CYCS
Uncontrolled tumor growth and invasion	hsa-miR-16-5p hsa-miR-17-5p hsa-miR-20a-5p hsa-miR-34a-5p	E2F3
Apoptosis—Bcl2 pathway—drug resistance	hsa-miR-16-5p hsa-miR-17-5p hsa-miR-20a-5p hsa-miR-34a-5p	MCL1
Promotion of tumor growth—drug resistance—poor survival	hsa-miR-16-5p hsa-miR-17-5p hsa-miR-34a-5p hsa-miR-106a-5p	MFN2
Cancer cell growth and survival—drug resistance—poor survival	hsa-miR-16-5p hsa-miR-17-5p hsa-miR-20a-5p hsa-miR-34a-5p	MYC
p53 pathway—proliferation and energy metabolism	hsa-miR-20a-5p hsa-miR-16-5p hsa-miR-106a-5p hsa-miR-223-5p	RNF115
Cell cycle deregulation and cell proliferation—drug resistance	hsa-miR-20a-5p hsa-miR-16-5p hsa-miR-34a-5p hsa-miR-223-5p	SENP1
Cellular transformation and growth	hsa-miR-16-5p hsa-miR-20a-5p hsa-miR-16-5p hsa-miR-34a-5p hsa-miR-106a-5p	SLC1A5
C-MYC pathway—poor survival	hsa-miR-16-5p hsa-miR-20a-5p hsa-miR-16-5p hsa-miR-106a-5p	SLC6A4
Ferroptosis—tumor progression	hsa-miR-20a-5p hsa-miR-16-5p hsa-miR-106a-5p hsa-miR-223-5p	SLC7A11
Cancer progression—EGFR/PI3K/AKT or MAPK/ERK signaling pathways	hsa-miR-20a-5p hsa-miR-16-5p hsa-miR-34a-5p hsa-miR-106a-5p	TMOD3

**Table 4:** Abbreviations, gene names, methods, and tissues on which the selected miRNAs were validated as targets from the miR Target Link Human database.

Abbreviation	Gene Name	Methods	Tissues	References (PMID)
BCL2	BCL2 Apoptosis Regulator	Luciferase reporter assay, qRT-PCR, Western blot, reporter assay, proteomics analysis, immunohistochemistry, microarray, sequencing, HITS-CLIP, immunoblot, immunoprecipitation	Cervix cells, gastric cells, bone cells, marrow cells, spleen, liver, kidney, lymph node, tracheal/bronchial epithelial cells, breast cells, ovary cells, embryonic kidney cells, gastric cancer cells, B cells, mesothelial cell, glioma cells	17877811 18449891 18362358 17351108 17707831 20643754 20876285 19269153 16166262 19903841 20371350 23907579 22473208 24148817 25435430 26397135 26722459
CYCS	Cytochrome C, Somatic	Proteomics, PAR-CLIP, PCR array	Breast cells, brain and liver	18668040 23446348 28097098
E2F3	E2F Transcription Factor 3	CLASH. HITS-CLIP, luciferase reporter assay, Western blot	Human embryonic kidney cells, B cells, stem cells	23622248 22473208 17252019
MCL1	MCL1 Apoptosis Regulator, BCL2 Family Member	HITS-CLIP, microarray, immunohistochemistry, luciferase reporter assay, qRT-PCR, Western blot, PCR array	Human embryonic kidney cells, leukemic cells, liver	22473208 18362358 23594563 28097098
MFN2	Mitofusin 2	Proteomics, luciferase reporter assay, Western blot, CLASH	Breast cells, lungs, human embryonic kidney cells	18668040 27640178 23622248
MYC	MYC Proto-Oncogene, BHLH Transcription Factor	TRAP, Western blot, CLASH, Luciferase reporter assay, Western blot, reporter assay; Western blot, qRT-PCR, microarray; sequencing.	Bone cells, mouse embryonic fibroblasts, breast cells, kidney cells, cervical cells, human fibroblasts, oral epithelium, stem cells, lymphoblastoid cells, bladder cells	24510096 18695042 23622248 19696787 21294122 21297663 22159222 20371350 25572695
RNF115	Ring Finger Protein 115	HITS-CLIP	Kidney cells, cervical cells, neuroblastoma cells, mouse embryonic fibroblasts, glial cells	23313552 23824327
SENP1	SUMO Specific Peptidase 1	HITS-CLIP, MIRT025428, PAR-CLIP	Human embryonic kidney cells	22473208 20371350 21572407
SLC1A5	Solute Carrier Family 12 Member 6	HITS-CLIP	Neuronal mouse cells, mouse primary embryonic fibroblasts	23313552
SLC6A4	Solute Carrier Family 6 Member 4	qRT-PCR, Western blot, HITS-CLIP	Lungs, brain	22940131 23313552 23313552
SLC7A11	Solute Carrier Family 7 Member 11	PAR-CLIP HITS-CLIP	Cervix cells, mouse neural progenitor cells, human retinal epithelial cells, primary mouse embryo fibroblasts, human embryonic stem cells, kidney cells	23313552 22012620 20371350 21572407
TMOD3	Tropomodulin 3	HITS-CLIP Proteomics	Cervical cells, colorectal cells	23313552 21566225

## Data Availability

Data are contained within the article.
